# Sarcopenia worsens overall survival following robotic esophagectomy for esophageal cancer

**DOI:** 10.1038/s41598-025-00058-7

**Published:** 2025-05-05

**Authors:** Jennifer Merten, Nabila Gala Nacul Mora, Jens Peter Hoelzen, Mazen Juratli, Andreas Pascher, Ann-Kathrin Eichelmann

**Affiliations:** 1 Department of General, Visceral and Transplant Surgery, University Hospital of Münster, Waldeyerstraße 1, 48149 Münster, Germany; 2 Department of Radiology, University Hospital of Münster, Münster, Germany

**Keywords:** Sarcopenia, Esophageal cancer, Esophagectomy, Robot-assisted minimally invasive esophagectomy (RAMIE), Minimally invasive esophagectomy (MIE), Long-term outcome, Gastroenterology, Oncology, Risk factors

## Abstract

Sarcopenia is a recognized independent risk factor associated with poor outcomes in cancer patients undergoing surgery. Patients with esophageal cancer are particularly susceptible to sarcopenia due to multiple factors. Purpose of the current study was to investigate the effect of sarcopenia on outcome and survival in patients undergoing full-robotic esophagectomy. This study includes all patients who underwent full-robotic abdominothoracic esophagectomy for esophageal cancer between January 2019 and December 2022. The skeletal muscle index, assessed by the preoperative computed tomographic staging scan, was used to classify the study cohort into a sarcopenic and a non-sarcopenic group. A total of 206 cases were included. With 168 patients (82%), prevalence of sarcopenia was high in the study population. The proportion of older (65.3 vs. 60.7 years, *p* = 0.0115), male (86% vs. 72%, *p* = 0.0469) and patients with tumor stenosis and/or dysphagia after completion of neoadjuvant therapy (71% vs. 44%, *p* = 0.0035) in the sarcopenic group was significantly higher than in the non-sarcopenic group. Sarcopenia did not affect short-term outcomes including complication rates. However, overall- (17.4 vs. 22.8 months, *p* = 0.0458) and disease-free survival (15.3 vs. 22.6 months, *p* = 0.0069) was significantly reduced in sarcopenic patients. Preoperative sarcopenia was not associated with altered short-term outcomes but reduced overall- and disease-free survival. These findings underscore the critical need for prehabilitation and nutritional support for sarcopenic patients undergoing full-robotic esophagectomy, a complex procedure with inherently high morbidity.

## Introduction

Sarcopenia, the progressive loss of skeletal muscle mass and strength, poses a significant challenge in the management of cancer. Esophageal cancer patients are especially vulnerable to sarcopenia due to several factors. The disease itself often leads to dysphagia and poor nutritional intake, which are compounded by the catabolic effects of the tumor and systemic inflammation. Moreover, esophageal cancer is an aggressive malignancy often treated with a combination of radio- chemotherapy, and surgery. Neoadjuvant therapy, while critical for downstaging the tumor, can contribute to muscle wasting through treatment-related side effects such as nausea, vomiting, anorexia, and fatigue^1^. These effects lead to reduced caloric intake and physical inactivity, accelerating the loss of muscle mass and negatively impact patient health and recovery^2,3^.

The presence of sarcopenia in esophageal cancer patients has been associated with a range of negative clinical outcomes. The diminished physiological reserve in these patients makes it harder for them to withstand the physical demands of major surgery, such as esophagectomy, which is known for its complexity and high morbidity. Sarcopenic patients are more likely to experience severe toxicity from chemotherapy, increased surgical complications, and longer hospital stays^4–7^. Furthermore, sarcopenia is linked to lower overall and recurrence-free survival rates in esophageal cancer patients^5,8^.

Since esophageal cancer patients inherently face poor outcomes, it is of great importance to identify sarcopenic patients before initiating therapy. Detecting sarcopenia early allows for targeted interventions, such as nutritional support and exercise programs, which can improve treatment tolerance, reduce complications, and enhance recovery, ultimately leading to better overall outcomes.

A key component aimed at improving surgical outcomes was the establishment of minimally invasive procedures. In this regard, robotic-assisted minimal invasive esophagectomy (RAMIE) has emerged as a cutting-edge approach at high-volume centers. RAMIE has been associated with fewer complications, reduced blood loss, shorter hospital stays, and quicker recovery, making it a promising advancement in the surgical treatment of esophageal cancer^5,9–11^. The available literature on sarcopenia and esophageal cancer has so far primarily addressed outcomes following minimally invasive procedures, and large number of these studies include Asian populations. To date, there is to the best of our knowledge no data that investigates the effect of sarcopenia in a RAMIE cohort. Knowing the negative effect of sarcopenia on surgical outcome as described above, the purpose of the current study was to investigate the impact of sarcopenia on postoperative complications and survival in patients undergoing RAMIE for esophageal cancer in a Western cohort.

## Methods

### Study population

This study includes all patients who underwent full-robotic abdominothoracic esophageal resection because of esophageal cancer between 01/2019 and 12/2022 at a high-volume tertiary care hospital. In detail, inclusion criteria were: (1) > 18 years, (2) elective full-robotic Ivor Lewis or McKeown esophagectomy, (3) histology type of adenocarcinoma or squamous cell carcinoma, and (4) complete data sets of computed tomographic images.

The diagnostic pathway included an endoscopy with endoscopic ultrasound (EUS) and biopsy, computed tomographic imaging and evaluation through a multidisciplinary cancer board with initiation of neoadjuvant therapy (CROSS or FLOT) according to current guidelines^12^. 3 weeks after completion of neoadjuvant therapy and re-staging imaging, a follow-up endoscopy was performed in our department to reassess the tumor for the planned esophagectomy. Surgery, performed 6–8 weeks after completion of neoadjuvant treatment, was performed as described earlier^13^.

Data were obtained on patient demographics, comorbidities, neoadjuvant treatment, histologic findings, postoperative course and length of stay. Overall (OS) and disease-free survival (DFS) was calculated from time of surgery until patient death, disease recurrence, or loss to follow-up as of February 2024.

### Ethics

All procedures performed in this study were in accordance with the ethical standards of the institutional research committee (Ethikkommission Münster, 2018–137-f-S) and with the 1964 Helsinki declaration and its later amendments or comparable ethical standards. Moreover, informed consent was obtained from all participants included in the study.

### Identification of sarcopenic patients

We used the skeletal muscle index (SMI), a key marker to assess sarcopenia, for identification of sarcopenic patients. The SMI was assessed using the preoperative computed tomographic re-staging scans by measuring the cross-sectional muscle area at the third lumbar vertebral level. According to Prado et al., who validated SMI cut-off values in a Western population, following cut-off SMI values were used to define sarcopenia: 52.4 cm^2^/m^2^ for males and 38.5 cm^2^/m^2^ for females^14^. Based on these cut-off values, the study population was divided into a sarcopenic and a non-sarcopenic group.

### Statistical analysis

All data are presented as means with standard deviation or median with range unless otherwise stated. Statistical analysis was performed with PRISM 10 for macOS (GraphPad Software, Inc. 2019) by using one-way ANOVA followed by a Holm-Sidak test for multiple comparisons and Fisher’s exact test for categorical variables. Kaplan–Meier method with log-rank tests was used for assessment of OS and DFS. Values with a *p* < 0.05 were considered statistically significant.

## Results

### Patient baseline characteristics

During the study period, a total of 206 patients were included. The vast majority were male (n = 171, 84%) and suffered from an adenocarcinoma (n = 170, 83%) located in the distal esophagus/esophagogastric junction (n = 180, 88%). Nine out of ten patients received neoadjuvant therapy (n = 180, 88%) and almost the entire study cohort underwent RAMIE (n = 197, 97%).

Mean BMI was 26.6 kg/m^2^ and mean SMI was 43.1 cm^2^/m^2^. Pearson’s correlative analyses demonstrated a correlation between BMI and SMI (r = 0.606; *p* < 0.0001). Based on SMI cut-off values as described above, 168 patients (82%) were classified as having sarcopenia.

We observed that the proportion of men (86% vs. 72%, *p* = 0.0469) and older patients (65.3 years vs. 60.7 years, *p* = 0.0115) in the sarcopenic group was significantly higher than in the non-sarcopenic group. BMI in the sarcopenic group was 6 points lower than in the non-sarcopenic group (25.5 kg/m^2^ vs. 31.4 kg/m^2^, *p* < 0.0001). Otherwise, both groups were comparable in terms of ASA-score, preoperative findings and administration of neoadjuvant therapy.

24 patients of the total study population (12%) underwent primary surgery without prior neoadjuvant treatment. 17 patients of this subgroup (70%) were sarcopenic, indicating the high incidence of sarcopenia in esophageal cancer patients regardless of whether neoadjuvant treatment was initiated or not. In case of neoadjuvant therapy, the endoscopy performed three weeks after completion of chemo-/radiotherapy to reassess the tumor for the planned esophagectomy, showed that two thirds of all patients suffered from a tumor stricture and/or dysphagia because of radiation- or chemotherapy-associated mucositis (n = 135, 66%). In this regard, the proportion of patients with stricture and/or dysphagia in the sarcopenic group was significantly higher than in the non-sarcopenic group (71% vs. 44%, *p* = 0.0035). One third of the total study population received a jejunal feeding tube either preoperatively or intraoperatively (n = 67, 33%). There was no difference in number of patients with jejunal feedings tubes in both groups, however. See Table 1 for detailed patient baseline characteristics and perioperative findings.

**Table 1:** Patient baseline characteristics and perioperative findings.

	All patients	Non-Sarcopenia	Sarcopenia	*p*-value	
	n = 204	n = 36 (18%)	n = 168 (82%)		
Sex				0.0469	*
Male	171 (84%)	26 (72%)	145 (86%)		
Female	33 (16%)	10 (28%)	23 (14%)		
Age (mean in years, range)	64.5 (29.2–89.1)	60.7 (39.5–79.9)	65.3 (29.2–89.1)	0.0115	*
BMI (mean in kg/m^2^, range)	26.6 (13.7–42.4)	31.4 (21–42.4)	25.5 (13.7–41.9)	< 0.0001	****
SMI (cm^2^/m^2^, range)	43.1 (24.1–71)	54.4 (39.1–71)	40.7 (24.1–52.3)	< 0.0001	****
ASA-Score				0.6109	ns
1	5 (2%)	0	5 (3%)		
2	109 (53%)	23 (64%)	86 (51%)		
3	88 (43%)	13 (36%)	75 (45%)		
4	2 (1%)	0	2 (1%)		
Preoperative findings					
Histology				0.4605	ns
Adenocarcinoma	170 (83%)	32 (89%)	138 (82%)		
Squamous cell carcinoma	34 (17%)	4 (11%)	30 (18%)		
Tumour location				0.699	ns
Upper	2 (1%)	0	2 (1%)		
Middle	22 (11%)	5 (14%)	17 (10%)		
Lower + junction (AEG I/II)	180 (88%)	31 (86%)	149 (89%)		
uT				0.1621	ns
uT0	1 (0%)	0	1 (1%)		
uT1	25 (12%)	8 (22%)	17 (10%)		
uT2	39 (19%)	9 (25%)	30 (18%)		
uT3	134 (66%)	19 (53%)	115 (68%)		
uT4	4 (2%)	0	4 (2%)		
missing	1 (0%)	0	1 (1%)		
uN				0.9733	ns
Negative	63 (31%)	11 (31%)	52 (31%)		
Positive	109 (53%)	19 (53%)	90 (54%)		
x	31 (15%)	6 (17%)	25 (15%)		
Missing	1 (0%)	0	1 (1%)		
Neoadjuvant therapy				0.1501	ns
None	24 (12%)	7 (19%)	17 (10%)		
Yes	180 (88%)	29 (81%)	151 (90%)		
Chemotherapy	64 (36%)	15 (52%)	49 (32%)		
Radiochemotherapy	115 (64%)	14 (48%)	102 (68%)		
Stricture/Dysphagia after completion of n(R)CTX	135 (66%)	16 (44%)	119 (71%)	0.0035	**
Jejunal feeding tube				0.3304	ns
Yes	67 (33%)	9 (25%)	58 (35%)		
No	137 (67%)	27 (75%)	110 (65%)		
Surgical approach/RAMIE				0.6093	ns
Ivor-Lewis	197 (97%)	34 (94%)	163 (97%)		
McKeown	7 (3%)	2 (6%)	5 (3%)		

n = number of patients, BMI = body mass index, SMI = small muscle index, ASA = American Society of Anaesthesiologists, AEG = adenocarcinoma of esophagogastric junction, n(R)CTX = neo-adjuvant (Radio)Chemotherapy, RAMIE = Robot-assisted minimally invasive esophagectomy, ns = non-significant.

### Short-term outcome

There were no differences between both groups in terms of postoperative tumor stage, number of harvested lymph nodes and resection margins. Patients from both groups remained in hospital/the intensive (including intermediate) care unit for the same length of time. Incidence of anastomotic leakage was 18% in total with a higher proportion of insufficiencies in the sarcopenic group (20% vs. 11%). This did not reach statistical significance. The only difference we observed between the two groups was the incidence of chyle fistulas: while every fifth patient in the non-sarcopenic group suffered from chyle leakage, incidence war very low in the sarcopenic group (19% vs. 3%, *p* = 0.0013). 30 day mortality was with 1% very low. Summarized, we did not find any relevant differences except for incidence of chyle leakage between the two groups in terms of short-term outcome. Table 2 shows details of the short-term outcome.

**Table 2:** Postoperative findings and short-term outcome.

	All patients	Non-Sarcopenia	Sarcopenia	*p*-value	
	n = 204	n = 36 (18%)	n = 168 (82%)		
Postoperative tumour stage				0.4702	ns
(y)pT0	48 (24%)	6 (17%)	42 (25%)		
(y)pT1	39 (19%)	10 (28%)	29 (17%)		
(y)pT2	30 (15%)	5 (14%)	25 (15%)		
(y)pT3	87 (43%)	15 (42%)	72 (43%)		
(y)pT4	0	0	0		
N0	112 (55%)	22 (61%)	90 (54%)	0.7956	ns
N1	46 (23%)	6 (17%)	40 (24%)		
N2	31 (15%)	5 (14%)	26 (15%)		
N3	15 (7%)	3 (8%)	12 (7%)		
Harvested lymph nodes	31.5 (10–70)	32.3 (13–70)	31.4 (10–60)	0.6024	ns
Positive lymph nodes	4.1 (1–23)	4.5 (1–14)	4 (1–23)	0.6788	ns
R0	194 (95%)	34 (94%)	160 (95%)	0.2213	ns
R1	10 (5%)	2 (6%)	8 (5%)		
Length of stay (days)	22.1 (3–188)	22.6 (10–62)	21.9 (3–133)	0.8117	ns
Length of stay ICU (days)	6.4 (1–91)	5.6 (1–62)	6.6 (1–91)	0.65	ns
Complications					
Anastomotic leakage	37 (18%)	4 (11%)	33 (20%)	0.3397	ns
Pneumonia	36 (18%)	5 (14%)	31 (18%)	0.6342	ns
Chyle leak	12 (6%)	7 (19%)	5 (3%)	0.0013	**
30d mortality	3 (1%)	0	3 (2%)	> 0.9999	ns

n = number of patients, d = day, ns = non-significant, ICU = intensive care unit (including intermediate care).

### Long-term outcome

**Fig. 1:**
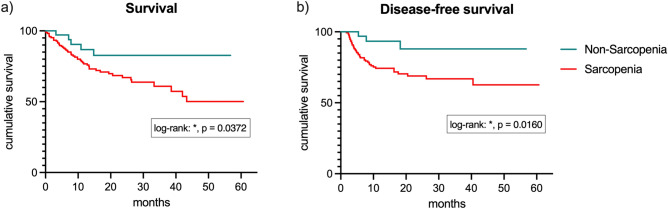
Survival curves comparing overall (**a**) and disease-free survival (**b**) in sarcopenic and non-sarcopenic patients.

The survival curves comparing overall and disease-free survival in sarcopenic and non-sarcopenic patients are shown in Fig. 1. Table 3 shows the absolute numbers for OS and DFS. In the follow-up period, three times as many patients died in the sarcopenic group than in the non-sarcopenic group (30% vs. 11%, *p* = 0.022). In both groups, main reason for death was recurrence (non-sarcopenic: n = 3/4, sarcopenic: 42/50). In this regard, three times as many patients suffered from recurrence in the sarcopenic group (25% vs. 8%, *p* = 0.0274). These numbers resulted in both reduced OS and DFS associated with sarcopenia: sarcopenic patients had an OS shortened by 5 months (17.4 vs. 22.8 months, *p* = 0.0458) and a DFS shortened by 7 months (15.3 vs. 22.6 months, *p* = 0.0069).

**Table 3:** Long-term outcome.

	All patients	Non-Sarcopenia	Sarcopenia	*p*-value	
	n = 204	n = 36 (18%)	n = 168 (82%)		
Death during follow-up	54 (26%)	4 (11%)	50 (30%)	0.022	*
Survival (in months)	18.4 (0–61)	22.8 (1–56.8)	17.4 (0.1–60.7)	0.0458	*
Recurrence	45 (22%)	3 (8%)	42 (25%)	0.0274	*
Disease-free survival (in months)	16.6 (0–61)	22.6 (1–56.8)	15.3 (0.1–60.7)	0.0069	**

## Discussion

In this study, we analyzed the impact of sarcopenia on postoperative complications and survival in patients undergoing RAMIE for esophageal cancer. Our patient characteristics reflect the typical profile of a Western esophageal cancer cohort, where male gender, histological subtype of adenocarcinoma, and age > 65 years are predominant^15,16^. With four out of five patients being sarcopenic prior to esophagectomy, we observed a very high number of malnourished patients. Furthermore, patients with sarcopenia had significantly poorer long-term and disease-free survival compared to non-sarcopenic individuals.

Sarcopenia is a significant concern among esophageal cancer patients, characterized by the progressive loss of skeletal muscle mass and strength. Esophageal cancer patients are especially vulnerable to sarcopenia due to factors such as tumor-related metabolic changes, malnutrition because of dysphagia, and treatment side effects. Therefore, the high prevalence of sarcopenic patients in our cohort is not surprising and is in line with findings from other studies, which report ranges of 14.4–80%^17^. The availability of preoperative CT scans facilitates the rapid and accurate determination of SMI, which is considered as gold standard test for noninvasively assessing skeletal muscle and sarcopenia and which allows early identification of sarcopenic patients^18^. However, a significant challenge arises from the variability in cut-off values employed by different authors in their studies, leading to inconsistent classifications of sarcopenia across populations and the broad range of reported incidences mentioned above^19^. In our study we used the cut-off value according to Prado^14^, the most frequently reported parameter and cut-off value^16^. Establishing standardized SMI cut-off values is essential for accurately identifying sarcopenic patients and ensuring the reliability of outcomes in clinical practice.

Sarcopenia is influenced by several risk factors, with age and male gender being the most significant in our study. It is well-known that older people are more likely to develop sarcopenia: patients experience a natural decline in muscle mass and strength, often exacerbated by decreased physical activity, hormonal changes, and chronic inflammation. This age-related muscle loss makes older adults particularly vulnerable to sarcopenia^20^. The data regarding gender as a risk factor for sarcopenia remain unclear and somewhat contradictory. While some studies suggest that female gender may predispose individuals to a higher risk of developing sarcopenia, possibly due to hormonal changes associated with menopause and lower muscle mass^21^, other research indicates that males may be at greater risk, potentially due to differences in body composition and lifestyle factors^22^. We also observed that two thirds of all patients suffered from a tumor stricture and/or dysphagia because of radiation- or chemotherapy-associated mucositis after completion of neoadjuvant therapy prior to surgery. In this regard, the proportion of patients with complaints was significantly higher in the sarcopenic group, suggesting that patients with an insufficient clinical response to neoadjuvant therapy, particularly those with persistent tumor stenosis, as well as those who struggle with therapy-associated side effects that impair adequate nutritional intake, are at higher risk for developing sarcopenia.

### Short-term outcome

Sarcopenia is a critical factor influencing surgical outcomes in cancer patients. Research has shown that sarcopenic patients often experience poorer surgical outcomes, including higher rates of morbidity and mortality^23^. For esophageal cancer patients undergoing esophagectomies, it has been demonstrated that sarcopenia is associated with higher rates of surgical morbidity and increased postoperative complications^8,16^, including respiratory complications^19^, which can significantly impact their recovery trajectory. In our study, we observed a trend towards a higher incidence of anastomotic insufficiencies in the sarcopenic group, but this remained statistically insignificant. The overall anastomotic leakage rate of 18% is within the expected range described in literature^24^ but still appears high. This is justified by the fact that our classification system, similar to the grading system of Müller et al.^25^, includes even minimal dehiscence of the stapled anastomosis as a “leak”. Variations in criteria—such as the threshold for what constitutes an insufficiency, including the extent of dehiscence or other complications—can lead to discrepancies in reported rates across studies. All insufficiencies were treated promptly with endoscopic vacuum therapy (EVT). This early and aggressive intervention follows the “failure to rescue” principle, aiming to quickly manage complications before they lead to more severe outcomes. By addressing insufficiencies at an early stage, we seek to minimize the impact of these complications. This proactive and aggressive management approach helped prevent complications from worsening, ultimately contributing to the low mortality rate of 1% observed in our patient population. EVT, along with complications such as pneumonia, may have contributed to the extended hospital stay. However, it is important to note that patients were only discharged once they had successfully re-established oral intake. This careful approach ensured that patients were nutritionally stable before leaving the hospital.

Our results did not demonstrate a worsened short-term outcome for sarcopenic patients, a finding which is contradictory to the studies mentioned above. One possible reason for this could be the introduction of a standardized nutritional protocol at our center. Additionally, one-third of the sarcopenic patients had a PEJ, allowing for enteral nutrition immediately after surgery. This approach may have contributed to improved postoperative outcomes despite the presence of sarcopenia. Furthermore, it should be noted that available literature includes patients undergoing traditional open esophagectomy or minimally invasive procedures (for example VATS, MIE, or hybrid reviewed by Park et al.^16^; the study of Mann et al. includes MIE and RAMIE patients^26^). To date, there is to the best of our knowledge no data that investigates the effect of sarcopenia prior to surgery in a full RAMIE cohort. Whether the robotic approach can truly counteract the negative short-term effects of sarcopenia is challenging to ascertain from this single-center study. The absence of data specific to RAMIE limits the understanding of how robotic-assisted techniques may influence the relationship between sarcopenia and surgical outcomes, emphasizing the need for further research in this area.

The only difference we could identify between both groups was the higher incidence of chyle leakage in the non-sarcopenic group, a contrary finding to what has been described in literature. Here, a reduced preoperative BMI (mostly BMI < 25 kg/m^2^) has been identified as a risk factor for developing chyle leakage after esophagectomy^27–29^. The relatively high incidence of chyle leakage described in literature (the ROBOT trial for example reports an incidence of chylothorax in 32%) reflects the radical nature of the oncologic surgery including en-bloc esophagolymphadenectomy with thoracic duct resection^10^. The observed increased incidence of chyle leakage in the non-sarcopenic group following radical lymphadenectomy is potentially due to the greater volume of intraabdominal and intrathoracic fat compared to individuals with lower BMI, which can obscure lymphatic vessels and make them more challenging to visualize during surgery.

### Long-term outcome

We observed a significantly reduced overall- and disease-free survival for sarcopenic patients undergoing RAMIE. In both groups, main reason for death was recurrence. Numerous meta-analyses have shown that esophageal cancer patients with sarcopenia undergoing MIE face significantly reduced overall and disease-free survival rates^8,16,19,30,31^. Fang et al. for example described in his meta‐analysis a significant association between sarcopenia and OS (hazard ratios (HR): 1.68, 95% confidence interval (CI): 1.54 – 1.83, *p* = 0.004, I 2 = 41.7%) and DFS (HR: 1.97, 95% CI 1.44 – 2.69, *p* = 0.007, I 2 = 61.9%)^8^. This association underscores the detrimental impact of sarcopenia on long-term prognosis in this vulnerable population although the exact reasons are not yet fully understood. Hypotheses suggest that sarcopenia is linked to impaired nutritional status, weakened immune function, and systemic inflammation, all of which negatively impact cancer outcomes. Studies have shown for example a significant correlation between sarcopenia and an elevated neutrophil/lymphocyte ratio, suggesting a weakened lymphocyte-mediated immune response to cancer^32,33^. Additionally, sarcopenia is associated with systemic inflammatory markers, which play key roles in carcinogenesis^34^, and is closely related to insulin resistance, a condition also tied to cancer initiation and progression^35^. In patients receiving (neo)adjuvant treatment, sarcopenia may increase the risk of therapy-related toxicity, further exacerbating the risk of poor outcomes^36^. Our data now expand on this existing literature by including findings related to patients undergoing RAMIE. This highlights the importance of addressing sarcopenia in this patient population. The need for targeted strategies to counteract sarcopenia, such as nutritional interventions and prehabilitation, becomes even more critical to improve survival outcomes.

## Limitations

The main limitations of this study include its single-center design and lack of randomization. As a monocentric study, the findings may not be generalizable to other institutions or broader populations, potentially limiting the external validity of the results. Additionally, the non-randomized design of the study introduces the risk of selection bias, as patient characteristics and treatment pathways may differ from those in randomized controlled trials. Furthermore, the group size is not homogeneous. Achieving homogeneity in the groups is therefore challenging, as sarcopenia is as already stated a common comorbidity in an esophageal cancer patient population, and its inclusion in studies inevitably leads to a skewed distribution. Moreover, variability in SMI cut-off values across studies adds to the heterogeneity in the field, complicating comparisons of sarcopenia’s impact across different populations. These factors should be considered when interpreting the findings, and future research should aim to replicate these results in larger, multicenter, and randomized studies.

## Conclusion

Sarcopenia prevalence is notably high among esophageal cancer patients prior to surgery. Our findings identified risk factors for sarcopenia, including male gender, advanced age, and tumor stricture or dysphagia following neoadjuvant chemoradiotherapy. While sarcopenia did not affect short-term surgical outcomes, sarcopenic patients undergoing RAMIE showed significantly reduced overall and disease-free survival in the follow-up period. These findings emphasize the critical need for early identification and management of sarcopenia to improve long-term outcomes in affected patients, particularly in aging populations and male patients.

## Data Availability

All data generated and/or analyzed during this study are included in this published article.
